# Management of patent ductus arteriosus in preterm neonates ≤32 weeks: a national survey of physician practices in Indian NICUs

**DOI:** 10.3389/fped.2026.1746228

**Published:** 2026-03-31

**Authors:** Aayushi Singh, Rema Nagpal, Suprabha K. Patnaik, Shalini Tripathi, Umamaheshwari Balakrishnan, Nandini Nagar, Mohit Sharma, Pradeep Suryawanshi

**Affiliations:** 1 Department of Neonatology, Bharati Vidyapeeth (deemed to be University) Medical College, Pune, India; 2 Department of Pediatrics, King George’s Medical University, Lucknow, India; 3 Department of Neonatology, Sri Ramachandra Medical College and Research Institute, Chennai, India; 4 Department of Pediatrics, Cloudnine Hospital, Bengaluru, India; 5 Department of Pediatrics, Santosh University Santosh Medical College and Hospital, Ghaziabad, India

**Keywords:** Indian NICUs, management variation, paracetamol, patent ductus arteriosus, preterm

## Abstract

**Introduction:**

Patent Ductus Arteriosus is very common in preterm neonates ≤ 32 weeks gestation, and practice variations, particularly in high-income countries have been reported, with minimal reports from LMIC countries like India. We aim to determine the current physician practices prevalent in the management of preterm PDA in neonates ≤ 32 weeks, in India.

**Methods:**

This 25-question survey was sent to physicians working in in Level III, II, and I NICUs in India.

**Results:**

238 neonatal physicians responded to the survey (response rate 57.8%), revealing that 81.5% worked in Level III units. 60% respondents had access to an echo machine in the NICU, while 13.9% did not have an echo machine in their units. 39.9% physicians reported being formally trained in neonatal echocardiography/proficient through experience, whereas 20.2% did not perform neonatal echocardiography. 40.7% respondents reported that echocardiography was performed by adult/ paediatric cardiologists. 62.2% preferred an ‘early-symptomatic treatment' strategy, while 29.4% favoured an ‘early-targeted treatment’ approach for ELBW neonates. Paracetamol was the first-line drug choice for 80% respondents (intravenous preferred over oral), followed by oral Ibuprofen (16.4%). 57.6% clinicians repeated the same drug for the second course when needed, and a similar number performed an echo-reassessment after completion of the full treatment course. 68.9% attempted medical closure up to two times, whereas access to surgical closure was limited for 70.5% of physicians.

**Conclusions:**

Access to echocardiography machines has improved in the last few years in India, though, even now, less than half of our neonatal physicians are appropriately trained in performing neonatal echocardiography. Physicians prefer an ‘early symptomatic treatment’ strategy and use Paracetamol as the first-line management, which differs from international practices. Access to surgical options in Indian NICUs is limited. Though management approaches are similar among physicians, critical areas such as echo availability, access to advanced echo training, and surgical (device) closure of PDA still need improvement to ensure equitable and optimal care for neonates with HsPDA.

## Introduction

The presence of a patent ductus arteriosus (PDA) is physiologically essential for foetal life, but postnatally, ductal constriction may be delayed, in preterm neonates, particularly those <30 weeks gestation, who may have a 65% incidence of persistent ductal patency beyond day 4 (1). There is clinical heterogeneity in the management of PDA, and a major debate exists regarding the appropriate management of hemodynamically significant PDA (HsPDA) (2), which arises from the perceived association of HsPDA with adverse outcomes vs. the unnecessary risks of medication exposure. Not surprisingly, this controversy has led to a lack of standardized practice and variations in management amongst neonatologists/paediatricians.

A recent European survey (2022) highlighted the lack of consensus amongst neonatologists working in a Level III NICU on treatment of PDA (3) The study noted the absence of national guidelines, differing approaches to screening strategies, marked variability in diagnostic criteria for assessing hemodynamic significance, and differing treatment strategies. Multiple other studies have also revealed a practice variation among NICUs regarding the use of routine echocardiography, treatment of presymptomatic PDA, and approach to surgery (4–9). These surveys have been performed in the resource-rich high-income countries (HIC), where the challenges are different compared to the low- and middle-income (LMIC) countries like India.

There are inherent challenges in a country as diverse as India. While there is a large pool of trained neonatologists available in the country, neonatal health care is divided between public and private hospitals, with marked differences noted in the available resources in these centres. There are disparities with the universal availability of bedside-echocardiography machines, the absence of nationally-accredited training courses in echocardiography for neonatal physicians so far, leading to skill differences amongst physicians; poor access to paediatric cardiologists, non-availability of some of the suggested non-steroidal anti-inflammatory drugs (NSAIDs), like Inj. Indomethacin and Inj. Ibuprofen, and variable access to surgical options for PDA. We, therefore, aimed to determine the current physician practices prevalent in the management of preterm PDA, in Indian NICUs.

## Methodology

### Questionnaire development

This cross-sectional survey was conducted between October and November 2024, following institutional ethics approval. The 25-question survey questionnaire in multiple-choice format, was aimed to gather physician preferences in managing neonates ≤32 weeks gestational age (GA) with HsPDA. It was developed by the authors (AS, RN), based on current practices, and reviewed by other authors (SP, PS). An online survey tool (Google Forms—https://www.google.com/forms/about) was created, tested initially for content, feasibility, and consistency among the authors, then distributed to academic groups via social media with a brief background on the survey's purpose. Responses could only be submitted after answering *all* questions, with consent implied upon form completion. Data collected included: (i) baseline information of respondents and their institution (ii) NICU demographic data (number of neonates admitted ≤32 weeks), (iii) availability of an echocardiography machine on-site, (iv) diagnostic criteria used to determine HsPDA and monitoring strategies (respondents were expected to consider HsPDA based on their usual institutional or personal criteria, incorporating clinical and/or echocardiographic findings), and (v) preferred medical and surgical management options. After sending the initial survey, reminders were sent twice weekly, and the survey was closed after one month**.**

### Statistical analysis

Data was collected through Google forms which was then exported into excel sheet. The results of the survey were analysed using descriptive statistics with the SPSS V.29 software. All data points were in qualitative format and presented as numbers and percentages. The weighted scores/ weighted average was used to give a more accurate representation of the overall average, in preference, for the physician-preferred echocardiographic and clinical criteria. The preferences are assigned numerical values (5,4,3,2, and 1 point for 1st, 2nd, 3rd, 4th, and 5th preference respectively). Each data value was multiplied by its assigned weight; the products were then summed. Chi-square test was used to compare the responses of survey participants as per their training status and level of care provided, *p* < 0.05 was considered statistically significant.

## Results

### Baseline characteristics

238 out of a total of 412 neonatal physicians responded, to whom the questionnaire was sent (response rate 57.8%). These physicians, from 15 states across India, worked in 226 different NICUs. More than half (50.8%, *n* = 121) were experienced with >5 years clinical experience, while young physicians undergoing fellowship/neonatology speciality training made up a smaller, though significant proportion (22.7%, *n* = 54) (Table 1). The majority of these respondents worked in Level III units (81.5%, *n* = 194), and represented 182 NICUs. 73.5% (*n* = 175) physicians reported admitting fewer than 20 preterm infants with gestational age (GA) ≤ 32 weeks per month, while 6.7% respondents (*n* = 16) reported admitting >40 neonates with GA ≤32 weeks per month in their NICUs. 87.8% respondents worked in NICUs which admitted <10 extremely low birth weight (ELBW) neonates per month (Table 1).

**Table 1:** Demographic profile of respondents and the NICUs.

Category	Sub-category	Number of Respondents (*n* = 238)	Percentage (%)
Position of the respondent in their NICU	Junior Faculty in teaching hospital (Assistant Professor)	30	12.6
	Consultant Paediatrician/Neonatologist < 5 yrs experience	33	13.9
	Consultant Paediatrician/Neonatologist > 10 yrs experience	55	23.1
	Consultant Paediatrician/Neonatologist 5–10 yrs experience	45	18.9
	Fellow/ Neonatal speciality trainee	54	22.7
	Senior Faculty in teaching hospital (Professor/Associate Professor)	21	8.8
Level of care provided by the NICU of respondent physician (as per NNF)	Level I: Near term and term neonates, Newborn Nursery	15	6.3
	Level II: Level I + Neonates ≤1,500 g, Special Care	29	12.2
	Level IIIA: Comprehensive care <28 weeks & ELBWs	75	31.5
	Level III B: III A + Paediatric surgery	100	42
	Level III C: III B + Cardiac surgery	19	8
Preterms with GA ≤ 32 weeks admitted per month	<10/month	92	38.7
	10–20/month	83	34.9
	21–40/month	47	19.7
	> 40/month	16	6.7
Total number of ELBW babies admitted per month	< 5/month	133	55.9
	5–10/month	76	31.9
	11–20/month	18	7.6
	> 20/month	11	4.6

Level I: Basic neonatal care of healthy newborns.

Level II: Specialty care of preterm infants with birth weight <1,500 g.

Level III: Comprehensive neonatal intensive care of ELBW and <28 weeks neonates, with facilities for Ventilator support (III-A), Paediatric surgery (III-B), Cardiac surgery (III-C).

Levels of care are as per the accreditation norms of National Neonatology Forum of India (NNF).

ELBW, extremely low birth weight; GA, gestational age.

### Access to echocardiography & physician expertise in echo-diagnosis of PDA

#### Availability of echocardiography

**Figure 1:**
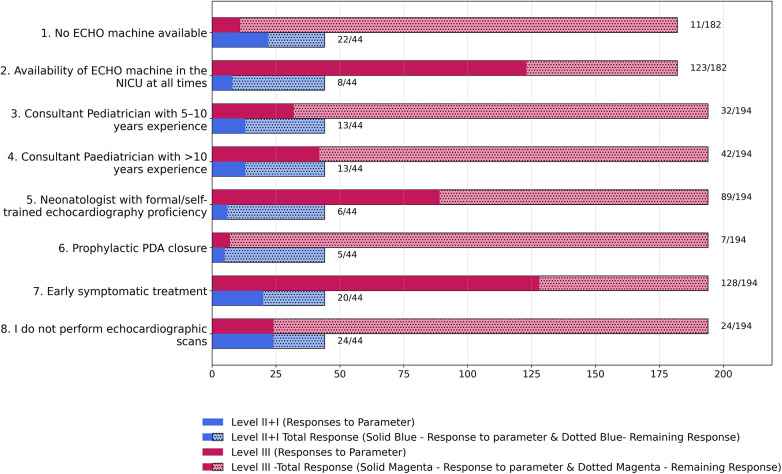
Key variations in management practices between level III and level II + I physicians. (Parameters 1 and 2 represent data from the NICU of the respondent physician).

60% of the respondents (*n* = 143) reported having an in-house echocardiography machine positioned (within the NICU), (Table 2), while 26% (*n* = 62) could access the machine within the hospital (but not in the NICU).13.9% (*n* = 33) of physicians did not have an echocardiography machine available in their hospital (Table 2). Ultrasound machines were not available in 50% Level II + I NICUs vs. 6.04% Level III NICUs (Level II + I −22/44 vs. Level III-11/182, *p* < 0.000001) (Figure 1).

**Table 2:** Access to echocardiography and physician expertise in echo-diagnosis of PDA.

Category	Sub-category	Number of Participants/NICUs (*n* = 238)	Percentage (%)
Availability of echocardiography machine	Available in the NICU at all times	143	60.1
	Available outside the NICU, baby needs transport to echo machine for evaluation	17	7.1
	Available outside the NICU but can be brought in as required	45	18.9
	There is no echo machine available in my hospital	33	13.9
Individual physician self-assessment in performing echo for PDA in smaller neonates	Neonatologist with formal or accredited training in echos	75	31.5
	Experienced Neonatologist, no formal training, regularly does echoes	20	8.4
	Fellows under training	55	23.1
	Paediatrician with some echocardiographic training	40	16.8
	I do not perform echocardiographic scans	48	20.2
Person usually performing/reporting echos for PDA (≤32 weeks) in respondent's NICU	Neonatologist with formal or accredited training in echos	64	26.9
	Experienced Neonatologist, no formal training but regularly does echocardiography	27	11.3
	Fellows under training	30	12.6
	Paediatric Cardiologist	62	26.1
	Adult cardiologist	35	14.7
	Echo is not performed in the unit	20	8.4

#### Physician expertise in performing the neonatal echocardiography

Less than half the physicians (39.9%, *n* = 95) reported either being formally accredited/ trained in neonatal echocardiography or proficiency acquired through experience, and independently performed echos; while 20.2% (*n* = 48) of the respondent physicians did not perform neonatal echocardiography at all. The remainder were either under training, performed under supervision or reported lower levels of skill (Table 2). As expected, of the above physicians well-trained in neonatal echocardiography, majority (89/95) worked in Level III NICUs (*p* < 0.0001). In response to a specific question on “person usually performing the echocardiography in the unit for neonates ≤32 weeks”, 91 respondents (38.2%), who were formally-trained or self-trained neonatologists, performed it themselves, while 97 respondents (40.7%) reported the echocardiography being done by adult/paediatric cardiologists (Table 2). 8.4% respondents indicated that echocardiography was not performed in their NICU.

### Diagnosis of PDA: clinical indicators and timing-to-first-echocardiography

#### Clinical criteria used to diagnose HsPDA

**Figure 2:**
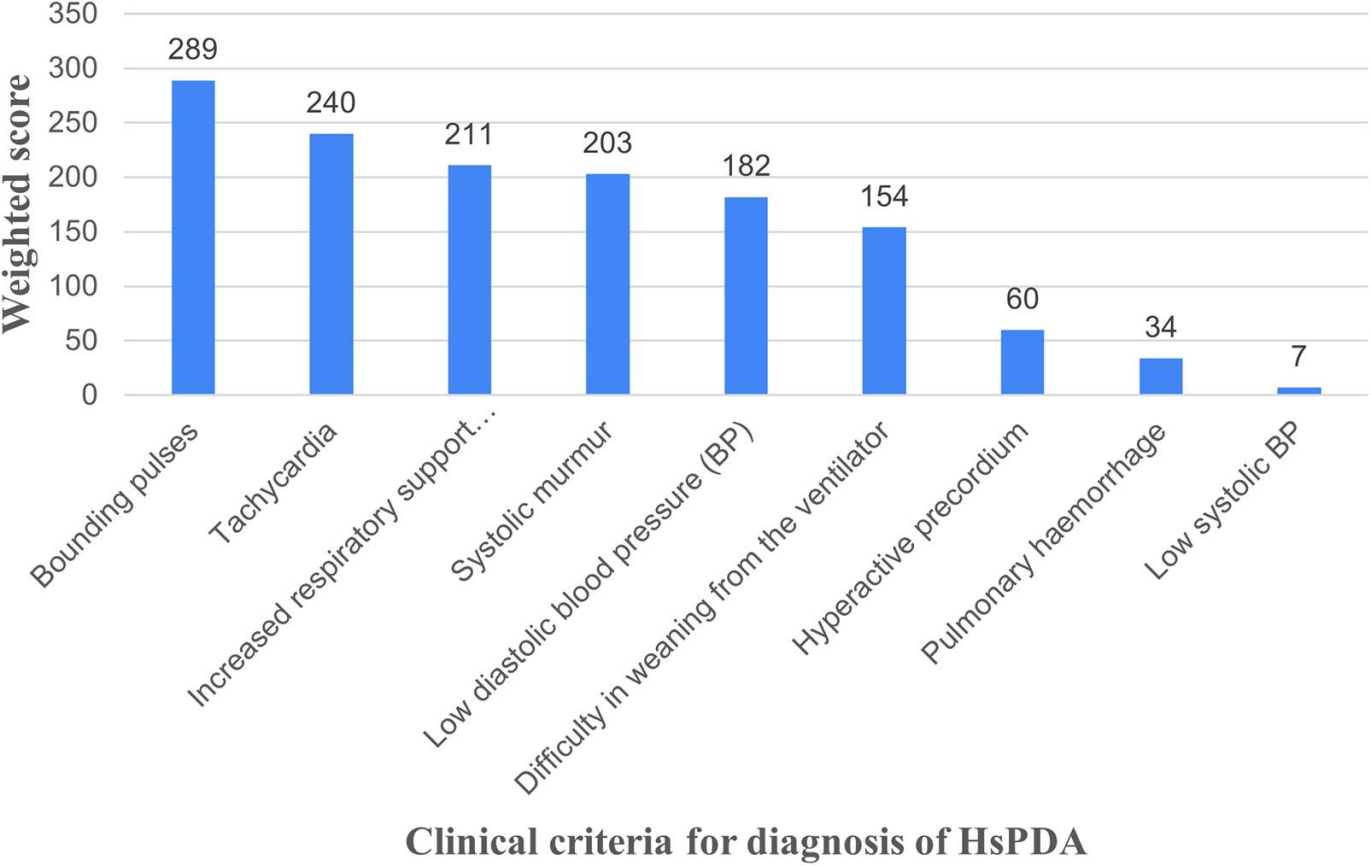
Weighted scores for physician preference for clinical criteria for HsPDA. (Weighted score: represents the overall average; each preference is assigned a numerical value e.g., 5,4,3,2 and 1 point for 1st, 2nd and subsequent preference respectively. Each data value was multiplied by its assigned weight and the products were summed.).

For the diagnosis of PDA, a majority of respondents (58.8%, *n* = 140) relied on a combination of clinical and echocardiographic criteria, while 28.2% (*n* = 67) used echocardiography alone. 11.8% (*n* = 28) physicians used only clinical criteria. Only 3 physicians (1.3%) incorporated biomarkers like Brain Natriuretic Peptide (BNP) alongside clinical and echocardiographic criteria. Figure 2 illustrates the ranked importance of clinical criteria, represented as weighted scores, considered as most diagnostic of PDA, of which bounding pulses were the most frequently chosen clinical criterion.

#### Timing of PDA scan

36.1% respondents performed echocardiography only when clinically indicated, and not routinely. 33.2% physicians performed “*routine scans*” between 24 and 72 h age, while 13.4% performed the scans ≤24 h of birth; while the rest felt *routine* scans were not necessary. On a specific question on the management strategy that would be followed if a neonate ≤28 weeks had echo-evidence of Hs PDA, without clinical signs, 57% were unlikely to treat the neonate with pharmacotherapy, while 43% were more likely to treat.

### Echocardiographic assessment of PDA significance

**Figure 3:**
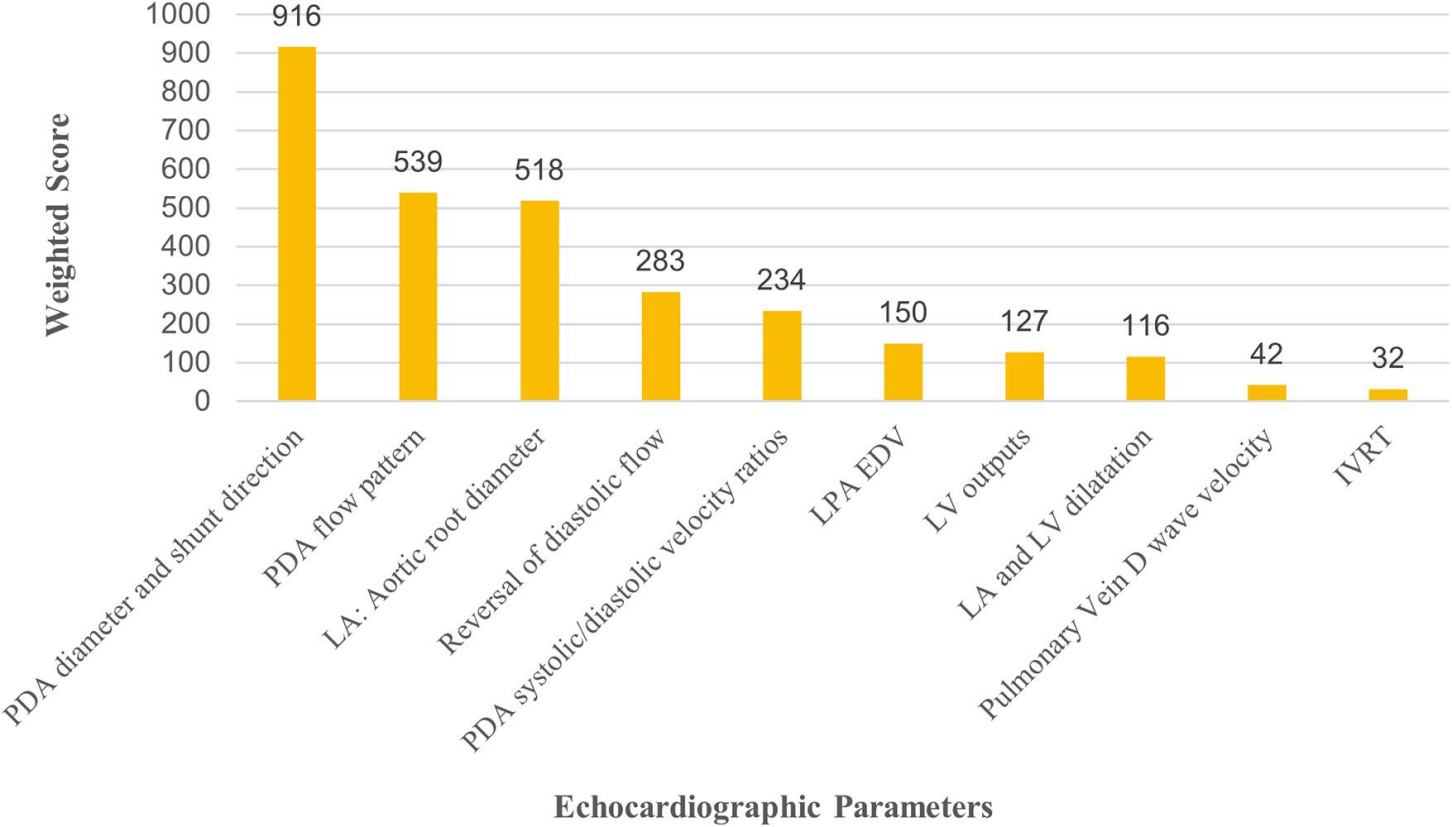
Echocardiographic parameters considered most significant for diagnosing hemodynamically significant PDA (HsPDA). *X* axis: Represents echocardiographic parameter. *Y* axis: Represents the weighted scores of each parameter, indicates clinician preference. IVRT, isovolumetric relaxation velocity; LA, left atrium; LPA EDV, left pulmonary artery end diastolic velocity; LV, left ventricle.

The following five were cited as the most important echo-criteria (from highest to lowest preference) in the diagnosis of HsPDA, and are represented as weighted-scores (Figure 3). PDA diameter and shunt direction had the highest overall preference (weighted-score 916), followed by PDA flow pattern (weighted-score 539), and other echo parameters. Only 58% physicians measured the ductal diameter on the grey scale.

### Medical management strategy

#### Medical pharmacotherapy

**Figure 4:**
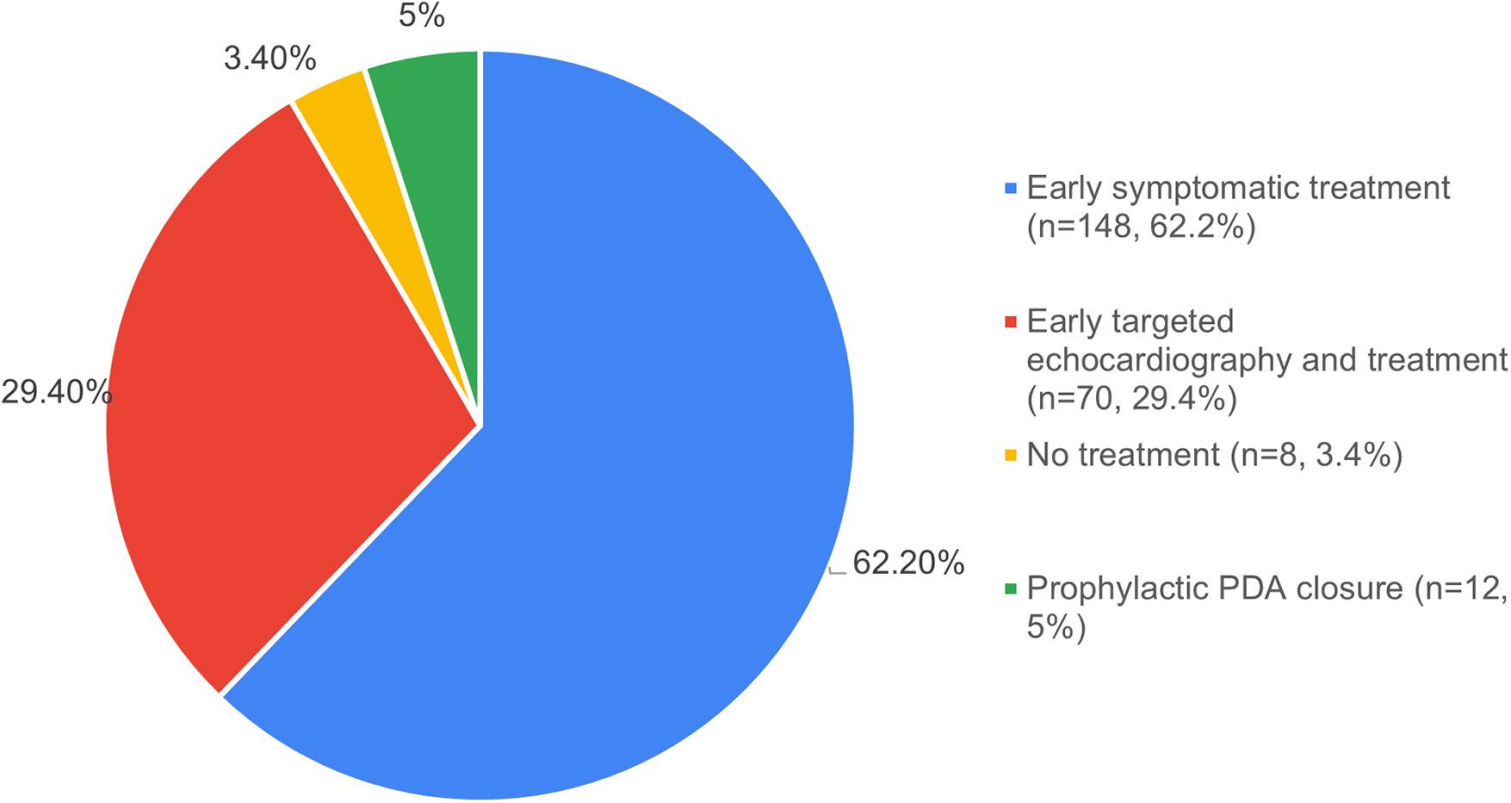
Preferred initial treatment strategy for HsPDA in ELBW neonates. (HsPDA, hemodynamically significant PDA; ELBW, extremely low birth weight).

**Figure 5:**
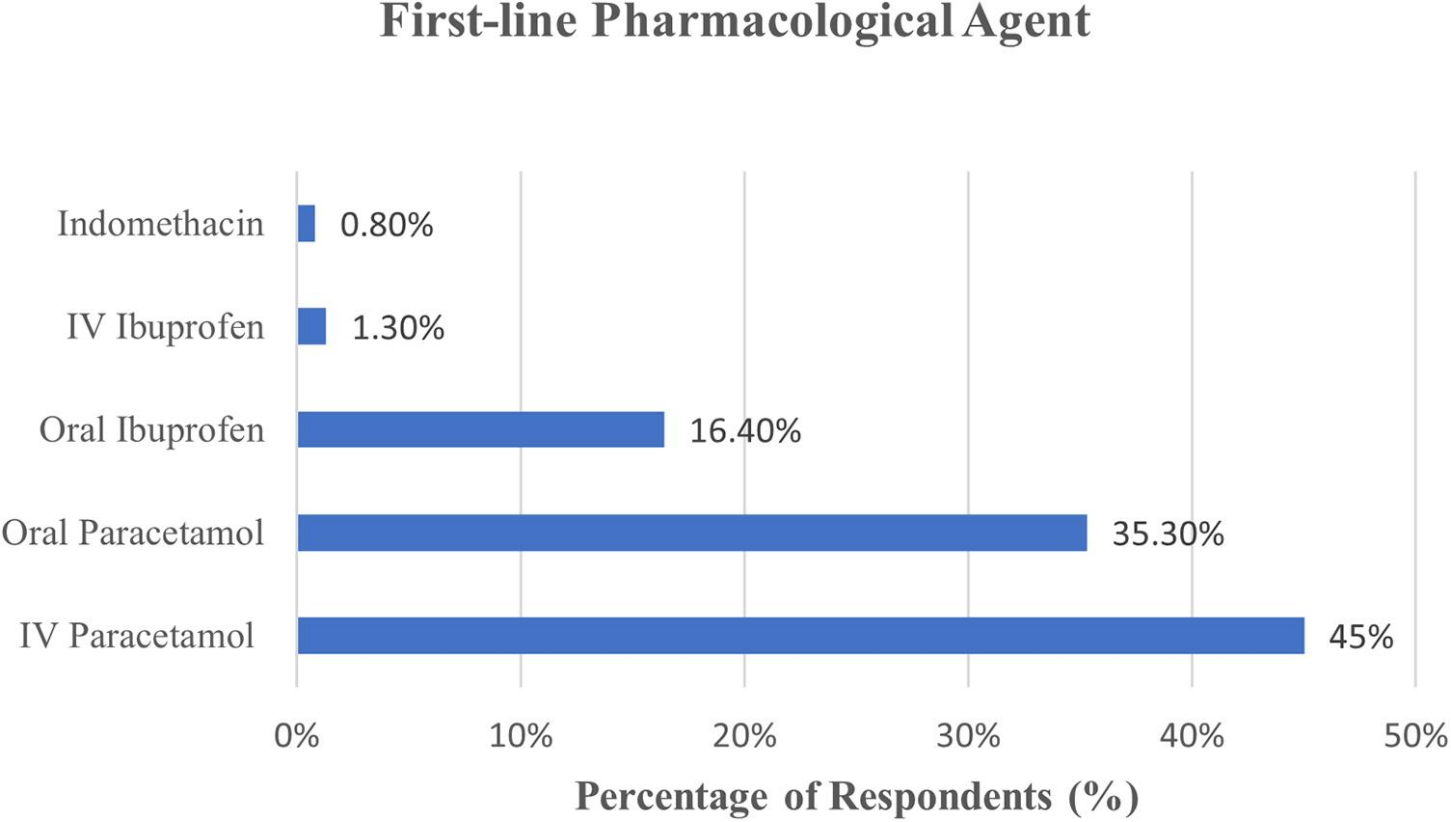
First-line pharmacological agents preferred for PDA closure.

62.2% (*n* = 148) respondents preferred an “*early-symptomatic treatment”* strategy (echocardiography shows HsPDA; neonate considered symptomatic due to PDA) for ELBW neonates, while 29.4% preferred an “*early-targeted treatment”* strategy (positive echocardiographic findings without clinical indicators) (Figure 4). Prophylactic treatment (treatment within 12 h of life, without knowledge of echo-status of PDA) was practiced by only 5.0% (*n* = 12). The preferred drug for a first-time ductal closure was intravenous (IV) Paracetamol/Acetaminophen (44.9%, *n* = 107), followed by oral Paracetamol (35.3%, *n* = 84) and oral Ibuprofen (16.4%, *n* = 39) (Figure 5). The preferred dosing for oral Ibuprofen was 10 mg/kg/dose (Day 1) followed by 5 mg/kg/dose (10–5–5) on the subsequent two days. Very few physicians used the alternate dynamic dosing schedules of Ibuprofen, namely the “15–7.5–7.5 mg/kg/dose” or the “20–10–10 mg/kg/dose” schedules.

#### Subsequent courses

On first-course failure/ductal reopening (after initial successful closure), 57.6% (*n* = 137) clinicians repeated the same drug for the second course, 35.3% (*n* = 84) chose an alternative drug, while 7.1% (*n* = 17) proceeded directly to surgical or device closure. 57.6% respondents (*n* = 137) performed an echo-reassessment after completion of the full treatment course, 18.1% (*n* = 43) opted to rescan the neonate after each individual dose, and 24.4% (*n* = 58) did not rescan the neonate at all, unless the clinical condition so warranted. The second course pharmacotherapy initiation, when necessary, was varied; given either by 12 h (13.9%, *n* = 33), 24 h (48.7%, *n* = 116), 48 h (21.8%, *n* = 52), or 72 h (15.5%, *n* = 37) after 1st dose completion.

The maximum number of pharmacological courses attempted for ductal closure was varied, with 68.9% (*n* = 164) preferring a maximum of two courses, while 27.7% (*n* = 66) preferred three courses. When HsPDA was detected beyond 1 month age, 42% physicians attempted closure with at least 1 course of medical pharmacotherapy, while 48.7% preferred to directly opt for surgical treatment.

#### Fluid restriction and clinical contraindications

Fluid restriction was routinely practised in neonates with HsPDA by 77.7% respondents. The “absolute contraindications to medical management” of PDA included persistent pulmonary hypertension (56.3% respondents), thrombocytopenia <50,000/mm³ (46.2%), serum creatinine >1.5 mg/dL (36.1%), severe intraventricular haemorrhage (IVH) (34%), and late PDA diagnosis beyond one month of age (31.1%).

### Surgical management

Access to surgical closure of PDA was limited for 70.5% (*n* = 168) respondents, who referred patients to higher centres for surgical (ligation or device closure) management, while 23.1%(*n* = 55) respondents reported availability of surgical facilities in their NICU. 6.3% physicians did not prefer any surgical management. On a specific question on strategy for duct closure if a neonate had HsPDA ≥1 month age, 49% preferred the surgical option. Amongst the surgical options (ligation vs. device closure), 63.8% physicians preferred device closure, of which 51.3% did not have the facility in their NICUs.

### Variations in management practices between levels of NICU care

Of the 238 respondents, 81.5% (*n* = 194) were from Level III units, representing 182 Level III units. 12.4% (24/194) level III physicians and 54.5% (24/44) of level II + I physicians did not perform echocardiography (*p* < 0.0001) (Figure 1). Prophylactic PDA closure was preferred by 3.6% (*n* = 7) Level III physicians, while 11.4% (*n* = 5) Level II + I physicians chose PDA prophylaxis for neonates ≤32 weeks (*p* 0.03). Other statistically significant differences between both levels included the presence of in-house echo machine in level III NICU compared to level II + I (*p* < 0.0001), more neonatologists formally and adequately proficient in neonatal echo in level III (*p* < 0.0001), more physicians not performing an echo in level II + I (*p* < 0.0001) (Figure 5).

## Discussion

The management of HsPDA in preterm infants, remains a subject of considerable debate and clinical heterogeneity. This cross-sectional survey, reflecting a wide spectrum of clinical experience, shows the within-country variations in management. We chose to conduct a physician survey as opposed to a unit-based survey, since we felt that the latter may not reflect on-ground management practices, given that PDA may be managed differently by different clinical teams on-service within the same NICU. Our study represents physician practices across different levels of NICU care, but predominantly Level III care. There are no national guidelines on management of PDA in preterm neonates, which probably explains the heterogeneity in clinical practice.

### Access to echocardiography and physician expertise

In this survey, 86% respondents had access to an echo-machine (in NICU or within the hospital), and 40% reported being proficient/formally accredited in neonatal echocardiography, both of which are an improvement over our previously published Indian survey (2019), when dedicated ultrasound machines were available in only 68% NICUs, 24% units reported inability to afford an ultrasound machine, and only 25% physicians reported having completed a structured/certified training course in point-of-care-ultrasound (POCUS) (10). This highlights the improved echo-accessibility and training standards in the last 5–6 years, and is likely to lead to improved echo-monitoring of sick neonates, and possibly improved outcomes, though these benefits remain to be studied. In comparison, 92% French NICUs (in 2011) were well equipped with an echo-machine (7), while the International Network for Evaluating Outcomes of Neonates (iNeo), a multinational collaborative of neonatal networks from 11 HIC countries in 2016, reported that 13%–100% units performed routine echocardiography, predominantly between 24 and 72 h after birth, with the exception of Japan, which performed all echos within 24 h of birth (6). Our survey also shows that 40% physicians relied on adult/paediatric cardiologists for the diagnosis of HsPDA, and implies the need for further training/accredited courses for the robust development of physician skill-sets in this specialised area. While 80%–100% neonatologists from Japan and Finland, and 60% Italian neonatologists perform echocardiography; <20% neonatologists perform echos in Canada, Israel, and Switzerland (6, 8), revealing a marked variation in international practice. A United States hemodynamic collaborative (2019) survey showed that in centres with a formal “Targeted neonatal Echocardiography (TnECHO) service”, neonatologists performed bedside-echocardiography; while in centres without the aforementioned service, surprisingly, only 72% had access to an echocardiography machine in their units, and furthermore, 74% respondents identified the need for high-quality echo-training programmes (5). We, therefore, emphasize the need for high-quality accredited training programmes, which should be an important part of the training of every neonatal physician, particularly in LMIC countries like India, with a burden of preterm birth, identified as 23.4% of the global preterm births (in 2014) (11).

### Diagnosis of PDA: clinical indicators and timing-to-first-echocardiography

#### Criteria utilised to diagnose HsPDA

The hemodynamic effects of an HsPDA include clinical findings, echocardiographic parameters, and serum biomarkers (12). Various authors have suggested that the decision to treat PDA depends on echocardiography and clinical signs that lead to hemodynamic effects (13–15), with the reported accuracy of clinical signs alone having low sensitivity (16, 17). Echocardiographic criteria tend to precede the development of clinical signs (17–20) by an average of 1.8 days (17), which is critical in smaller or sicker neonates. In our study, 58.8% of respondents preferred combined criteria to initiate PDA treatment, while 28.2% relied solely on echocardiography. Brissaud et al. found that 72.3% of units used combined criteria, whereas 27.6% relied only on echocardiographic criteria, regardless of the patient's clinical status (7). Notably, only 11.8% of physicians in our study believed in relying solely on clinical signs to determine HsPDA, compared to 13.9% of physicians without an echo-machine in their hospital, possibly indicating a growing recognition of the need for echo validation of clinical signs. There is low certainty-evidence regarding the accuracy of serum biomarkers like BNP in PDA, and their validation for local populations and outcomes is needed (21). 13% of Italian NICUs use BNP, but only 1% of our physicians utilize it, likely due to cost considerations (8).

#### Timing to scan for PDA

33% of respondents were more likely to “routinely scan” **neonates** ≤32 weeks, between 24 and 72 h. Other surveys (3, 6, 7) indicate that between 46%–84% of participants performed routine screenings mainly between 24 and 72 h, with Japan being an exception, conducting all echos within 24 h of birth (6).

#### Echocardiographic assessment of hemodynamic significance of PDA

Neonatal echo-assessments play a significant role in HsPDA-assessment (12). Our respondents preferred PDA diameter, shunt direction and flow patterns as markers of significance, very similar to Hundscheid et al. (3). French physicians considered ductal diameter (65%) (measured in colour doppler), LPA end diastolic flow (50%), and left-atrium-aortic-root-ratio (LA: Ao) (61%) amongst the 3 important criteria suggestive of HsPDA (7). Respondents with publications on PDA gave a higher ranking to diameter, transductal flow patterns and abnormal/retrograde diastolic flow (ductal steal) in the celiac trunk (3).

### Management strategies: medical management and echo-monitoring

A recent clinical guideline suggests that all the prostaglandin inhibitor drugs appear equally effective in PDA management (12). Our survey revealed that 80% respondents preferred Paracetamol/ Acetaminophen (IV over oral) more than oral Ibuprofen as the *first-line* pharmacotherapy for HsPDA. This is at variance with international practice, where Paracetamol is given as *first-line* pharmacotherapy in only 0%–23% of neonates (6, 8, 22, 23). We speculate that Paracetamol is preferred, possibly due to its presumed superior safety profile, even though both drugs (oral/IV Paracetamol and oral Ibuprofen) are readily available in India and have similar efficacy. Trends in PDA management from the NICHD neonatal network reveal the decrease in Indomethacin use (2012 to 2021, 21.6% to 8.6%, *p* < 0.0001); while the use of Paracetamol has increased (2016 to 2021, 1.6% to 12.9%, *p* < 0.0001), with an unchanged usage of Ibuprofen (4). The Pan American Hemodynamic collaborative (2019) (5) report preference for pharmacological treatment with Ibuprofen (75%), Indomethacin (11%), and Paracetamol (14%); while 77%–100% Italian NICUs used Indomethacin/Ibuprofen as first-line medications; while 0%–23% used Paracetamol as first-line (6). However, the use of Paracetamol has significantly increased over the years, particularly in cases of failure of ductal closure, and contraindications/complications with other medications. Its use has been reported in 65% of neonates < 27 weeks in French NICUs (24), 26% of neonates in the PDA-TOLERATE trial (25), and prescribed by 70%–80% of neonatologists in Australia, New Zealand (26), as well as 65% of UK physicians (22, 23). In Italian NICUs, 24% and 47% of neonates from 25 to 28 weeks GA, and 23–24 weeks GA respectively, are administered Paracetamol (27, 28). There is moderate-certainty evidence of little/no difference in effectiveness between Paracetamol and Ibuprofen, and low-certainty evidence in Paracetamol vs. other NSAID comparisons, but these studies were conducted in moderate preterm neonates, and efficacy/safety in ELBW neonates require further research (29). Our study questionnaire was more generic, and did not specifically ask questions on drug usage for ELBW neonates. We emphasise, however, that these findings for Paracetamol usage reflects physician preference rather than evidence-based superiority, and should not be interpreted as treatment recommendations.

Our respondents preferred IV Paracetamol over oral, followed by oral Ibuprofen. The dosage schedule for Paracetamol (15 mg/kg/dose, every 6 h for 3–5 days) and Ibuprofen (10–5–5 mg/kg/day for 3 days) aligns with international guidelines. Multiple studies report that the preferred dosing schedule for Ibuprofen is 10–5–5 mg/kg/day (3, 7, 8), preferentially for a 3-day course (8) which is associated with an 80% chance of ductal closure in neonates over 27 weeks, with higher doses needed for younger neonates (12, 30). Oral Ibuprofen appears to be more effective in PDA closure than IV Ibuprofen, with high-dose Ibuprofen being superior to standard doses (31). Not all NSAIDs are available in India; oral Ibuprofen and IV and oral Paracetamol are readily available and affordable, while Indomethacin is occasionally available, and IV Ibuprofen is not available.

In our survey, the contraindications to medical management of PDA included persistent pulmonary hypertension, thrombocytopenia <50,000/mm³, serum creatinine >1.5 mg/dL, and severe intraventricular haemorrhage (IVH). Other studies (7, 8) have demonstrated similar contraindications.

#### Prophylactic pharmacotherapy

The evidence for prophylactic interventions includes studies conducted within the first three days of life and one study on prophylactic surgical ligation in the pre-surfactant era (12). Mitra et al. note that prophylactic Indomethacin reduces symptomatic PDA (RR 0.44; 95% CI 0.38–0.500) and severe IVH (RR 0.66; 95% CI 0.53–0.82); prophylactic Ibuprofen reduces risk of PDA by day 3 (RR 0.39; 95% CI 0.31–0.48) and reduces severe IVH (RR 0.67; 95% CI 0.45- 1.00) (31), with an uncertain effect of prophylactic acetaminophen on severe IVH. In our study, overall, only 5% of physicians preferred prophylaxis for PDA, although more Level II + I physicians (11%) chose PDA prophylaxis for neonates ≤ 32 weeks. We speculate this was due to the challenges of transporting smaller neonates from peripheral NICUs to tertiary care centres, in the absence of a national neonatal emergency transport service/system. In comparison, the Italian survey found that no participating centre reported using prophylaxis as a strategy (8).

#### Subsequent courses of pharmacotherapy

In the case of 1st dose ductal closure failure, 57.6% of respondents in our survey repeated the same drug, while 35.3% switched to an alternative drug. A majority (62.6%) gave the medication by 24 h after cessation of the 1st course. Brissaud et al. noted that following the first-course failure, the time interval between two courses ranged from 1 to 3 days for 75.6% of the NICUs (7). Ficial et al. determined that if the second course failed, management options included surgical ligation (46%), a third course of pharmacological intervention (10%–23%), or “watchful waiting” (6%) (8). Our survey revealed that a majority (68.9%) respondents preferred a maximum of 2 courses of pharmacotherapy only. The failure rate for ducal closure with monotherapy with cyclooxygenase inhibitors or Paracetamol is high, especially in neonates < 28 weeks (32) and the effectiveness of combination therapy is under research, but was not interrogated in this study.

#### Monitoring during treatment

57.6% physicians rescanned neonates only after completing a course, unlike 93% of Japanese NICUs, which repeat an echo after each dose, while most NICUs from other countries repeat an echo after each course of pharmacotherapy (6). Although there are no guidelines for this practice, frequent rescanning may help with dose adjustment or discontinuation during treatment, potentially reducing drug-related side effects and representing an area for further research.

#### Fluid restriction

Fluid restriction is routinely practiced as supportive therapy by 77% of our physicians; meanwhile, an Italian survey showed that 47% NICUs used fluid restriction for all neonates with PDA, while 43% NICUs used it only in cases of HsPDA (8).

### Surgical management

Access to surgical closure of PDA remains a major limitation in India, reported by 70% of respondents, highlighting a significant infrastructure gap in advanced PDA care in Indian NICUs. Regarding the best strategy for neonates with previously undetected Hs PDA beyond 1 month of age, 49% of physicians preferred surgical management, favouring device closure over surgical ligation. In 2011, 39% of French NICUs considered surgery the first- line treatment in certain cases, such as contraindications to medical therapy or PDA malformations (7). A 2023 review of the global status of transcatheter PDA closure for ELBW babies by Sathanandam et al. noted that many high- income countries have adopted this approach, especially for neonates under 1 kg (9), suggesting it may have possibly replaced surgical ligation as the preferred method for definitive PDA closure. The benefit of transcatheter device closure over medical therapy is its ability to ensure definitive PDA closure while avoiding the risks associated with thoracotomy, particularly in high- risk neonates ≤ 26 weeks and under 1000 g (9). Although evidence in this area is growing, data from India on device closure remains limited (9). We found very few case reports on device closure in Indian neonates (33–35). Treatment trends for PDA procedural closure over the last nine years in U. S. academic hospitals show decreased rates of PDA ligation (8. 4% to 2. 1%, *p* < 0. 0,001) and increased rates of transcatheter closure (0. 8% to 4. 8%, *p* < 0. 0,001) (4). The Percutaneous Interventions vs. Observational Trial of Arterial Ductus in Low-Birth-Weight Infants (PIVOTAL; ClinicalTrials.gov NCT 05,547,165) is currently studying early transcatheter closure (within 7–10 days) vs. conservative management in neonates under 28 weeks gestation, with results pending (4). The limited access to these advanced interventions in India underscores the need for substantial investment in training and infrastructure to offer comprehensive PDA management options. The continued reliance on medical therapy, even for late- presenting PDAs as indicated by our survey, where 42.0% attempted medical management beyond one month of postnatal age, may be due to restricted access to surgical or transcatheter closure options.

### Strengths and limitations of the study

To the best of our knowledge, this is the first Indian survey conducted to identify physician-preferred practices in PDA management in NICUs across India. Our questionnaire focused on management approaches for preterm neonates ≤ 32 weeks GA, and to keep the survey short, did not include specific questions about PDA management for neonates < 26 weeks and < 750 g. Therefore, the results cannot be generalized to practices for this smaller group of preterm neonates. Additionally, since it is a physician-based survey, there is a possibility that individual physician preferences or practices may differ from those of the unit, and respondent bias could also influence the results. We also acknowledge that there could be an under-representation of remote or non-academic NICUs, leading to selection bias. Furthermore, due to the lack of standardized national guidelines for preterm PDA management, practices are often personalized, and a questionnaire might not fully capture all the nuances involved in PDA management.

## Conclusions

This survey reflects the preferred clinical practices among neonatal physicians in India, revealing that access to ultrasound machines in NICUs has significantly improved; less than half of our neonatal physicians are formally trained in performing neonatal echocardiography, though a notable improvement compared to before; physicians favour an “early symptomatic treatment” approach and a majority prefer Paracetamol as *the first-line* medication, which differs from international practices. The findings emphasize the need for improved, high-quality accredited neonatal echocardiography training programs and highlight the limited availability of surgical backup in Indian NICUs for PDA, especially device closure, the emerging surgical method. While management approaches are similar in many areas, some crucial gaps exist, where efforts to increase echo availability, enhance access to advanced echo training, and expand interventional cardiology services—particularly device closure—could ensure equitable and optimal care for neonates with HsPDA. Developing national guidelines or consensus protocols could help standardize clinical care.

## Data Availability

The original contributions presented in the study are included in the article, and any further inquiries can be directed to the corresponding author.
